# Small-Molecule Theranostic Probes: A Promising Future in Neurodegenerative Diseases

**DOI:** 10.1155/2013/150952

**Published:** 2013-11-12

**Authors:** Suzana Aulić, Maria Laura Bolognesi, Giuseppe Legname

**Affiliations:** ^1^Department of Neuroscience, Scuola Internazionale Superiore di Studi Avanzati (SISSA), Via Bonomea 265, 34136 Trieste, Italy; ^2^Department of Pharmacy and Biotechnology, Alma Mater Studiorum, University of Bologna, Via Belmeloro 6, 40126 Bologna, Italy

## Abstract

Prion diseases are fatal neurodegenerative illnesses, which include Creutzfeldt-Jakob disease in humans and scrapie, chronic wasting disease, and bovine spongiform encephalopathy in animals. They are caused by unconventional infectious agents consisting primarily of misfolded, aggregated, **β**-sheet-rich isoforms, denoted prions, of the physiological cellular prion protein (PrP^C^). Many lines of evidence suggest that prions (PrP^Sc^) act both as a template for this conversion and as a neurotoxic agent causing neuronal dysfunction and cell death. As such, PrP^Sc^ may be considered as both a neuropathological hallmark of the disease and a therapeutic target. Several diagnostic imaging probes have been developed to monitor cerebral amyloid lesions in patients with neurodegenerative disorders (such as Alzheimer's disease, Parkinson's disease, and prion disease). Examples of these probes are Congo red, thioflavin T, and their derivatives. We synthesized a series of styryl derivatives, denoted theranostics, and studied their therapeutic and/or diagnostic potentials. Here we review the salient traits of these small molecules that are able to detect and modulate aggregated forms of several proteins involved in protein misfolding diseases. We then highlight the importance of further studies for their practical implications in therapy and diagnostics.

## 1. Introduction

Neurodegenerative diseases are a medical, social, and economic problem of paramount importance in developed countries. Besides the fact that their etiology is generally unknown, developing therapeutic and diagnostic interventions for diseases of the central nervous system (CNS) is further complicated by the impermeability of the blood brain barrier (BBB). Thus, Alzheimer's disease (AD) and prion diseases are still not curable with drugs, and only in 2012 [1–3] positron emission tomography (PET) imaging probes have been included in the AD diagnostic armamentarium.

In recent years, the close cooperation between drug delivery/treatment and molecular imaging disciplines has resulted in a relatively new branch of knowledge, known as theranostics. The term theranostics was coined to indicate the concomitant therapeutic and diagnostic properties in a single agent. The purpose of theranostics is to optimize the efficacy and safety of therapy, as well as to streamline the entire drug development process. Several exciting examples of theranostic systems have now been reported in the literature for the treatment of cancer [4], atherosclerosis [5], and gene delivery [6], but very few examples are reported in the neuropathological field, especially in the prion field. In our recent work [7] we detailed the development of a small molecule with fluorescent properties that is able to simultaneously detect and inhibit A*β* and PrP^Sc^ plaques in *in vitro* studies. The progress to date in the design and utilization of these compounds is discussed herein. 

## 2. Human Prion Diseases 

The term prion (pronounced “pree-on”) is the acronym for proteinaceous infectious particle. The prion hypothesis was put forward in 1982 to explain the surprising transmission mechanisms of this unconventional protein [13, 14]. The discovery that proteins can behave like infectious agents to transmit disease is a milestone in biology. In fact, what sets prions apart, as proposed by Prusiner [14], is that the actual infectious principle consists merely of protein and is capable of replicating and transmitting infections without the need for informational nucleic acids. Over the past decade, there has been renewed interest in proteins causing neurodegeneration since they may all act as prions (i.e., amyloid-*β*, *α*-synuclein). This hypothesis has profoundly influenced the development of diagnosis methods and effective therapies for the corresponding diseases.

Prion diseases, also known as transmissible spongiform encephalopathies (TSEs), occur in both humans and animals. Prion diseases are a group of rapidly progressive disorders characterized by a defined spectrum of clinical abnormalities. The number of human and animal diseases recognized as TSEs has increased steadily in recent years. They all share similar hallmarks such as the spongiform degeneration of the brain and variable amyloid plaque formation (PrP^Sc^). In fact, PrP^Sc^ is the disease-associated isoform of the endogenously expressed prion protein (PrP^C^), which may be present as amyloid deposits. The first cases of human prion disease, Creutzfeldt-Jakob disease (CJD), were reported in the 1920s [15, 16]. Since then, different forms of human TSEs have been described that can appear as sporadic, inherited, or iatrogenic disorders; they include CJD, Gerstmann-Sträussler-Scheinker syndrome (GSS), fatal familial insomnia (FFI), and kuru. In animals, several TSEs have been reported, including scrapie in goats and sheep, bovine spongiform encephalopathy (BSE) in cattle, chronic wasting disease in deer and elk, and transmissible mink encephalopathy.

Here we review some crucial points of human prion disorders.

### 2.1. CJD

CJD presents as a sporadic, hereditary (familial), or acquired (iatrogenic or BSE-related) illness. Approximately 85% of all CJD cases occur sporadically, geographically, and ubiquitously, with an incidence rate of 0.5–2 cases per one million people per year. The median age of onset is the seventh decade (64 years) equally affecting men and women [17]. The clinical progression typically occurs over a few weeks. Around 70% of those afflicted die in less than 6 months. Typically, CJD presents with progressive dementia and cerebellar degeneration, characterized by spongiosis, neuronal loss, astrogliosis, and a clinical syndrome accompanied by dementia, memory loss, ataxia, and myoclonus. Early symptoms, present in approximately one-third of the cases, include fatigue, insomnia, depression, weight loss, headaches, general malaise, and ill-defined pain sensations. In addition to mental deterioration and myoclonus, frequent additional neurological features include extrapyramidal signs, cerebellar ataxia, pyramidal signs, and cortical blindness [18]. Homozygosity for methionine (Met) or valine (Val) at position 129 of the human PrP gene (*PRNP*) has been identified as a predisposing factor in the majority of sporadic and iatrogenic CJD cases [19, 20]. 

### 2.2. Familial CJD

Familial CJD (fCJD), representing 5–15% of all CJD cases, is classified into many haplotypes based on *PRNP* mutations present in the open reading frame and codon 129 on the mutant allele [21]. The majority of fCJD cases (>70%) have been associated with codon 200 mutations (E200K) [22–24] or with a codon 178 mutation (D178N) in the *PRNP *gene [25–27]. The symptoms of the familial form of CJD vary depending on the type of PrP mutation involved [28].

### 2.3. Iatrogenic CJD

Iatrogenic CJD (iCJD) is a very rare disease resulting from neurosurgery, corneal grafting, human dura mater implants, and the use of human growth hormone (hGH) and pituitary derived gonadotropin (hGNH). Iatrogenic CJD was first recognized in 1974 in a US patient who received a corneal transplant from a donor later proven to have died from CJD [29]. Worldwide, at least 226 cases of iCJD, including 26 US cases, have been associated with administration of contaminated human growth hormone (hGH) from cadavers. Of 74 UK cases reported from 1979 till 2011, 65 individuals received human-derived growth hormone, the other 8 individuals received infected dura mater implants, and one case of iCJD was reported after receiving human gonadotropin. In April 2013 one case of probable iCJD was reported (http://www.cdc.gov/eid) after treatment for 23 months with commercial cadaveric hGH when the patient was 6 years old. At the age of 33, 26.5 years (range 25.5–28 years) after the midpoint of commercial cadaveric hGH treatment, dizziness and gait imbalance developed, causing a fall. Seven months after the fall, he entered a state of akinetic mutism; he died 9 months after symptom onset. A small number of additional cases, known as variant CJD, are caused by secondary infection transmitted by transfusion of blood products. No new sources of disease have been identified, and current practices, which combine improved recognition of potentially infected persons with new disinfection methods for fragile surgical instruments and biological products, should continue to minimize the risk for iatrogenic disease until a blood screening test for the detection of preclinical infection is validated for human use [30].

### 2.4. Variant CJD

In 1995 and early 1996, a small number of remarkably young CJD patients were diagnosed in the United Kingdom. Due to its similarity to sCJD, this human disease was termed new variant of CJD (vCJD) [31]. In contrast to sCJD, the median age of onset of the disease in vCJD patients is 28 years (sCJD 64 years) and the clinical course is prolonged (median 14 months, sCJD 6 months). The appearance of vCJD in the United Kingdom and the experimental evidence that vCJD is caused by the same prion strain responsible for BSE raised the possibility of a vCJD epidemic [32, 33]. Laboratory transmission studies in transgenic mice showed that the characteristics of vCJD, including incubation period and neuropathological changes, are very similar in BSE and vCJD [34]. The favored hypothesis for transmission of BSE to humans is a dietary exposure to prion-contaminated bovine tissue (likely CNS) in the 1980s [35]. Variant CJD is difficult to distinguish from other neurological disorders, hence a definitive diagnosis has relied on neuropathology. It has been shown that vCJD can be diagnosed by PrP^Sc^ immunostaining on a tonsil biopsy [36]. The majority of vCJD cases have been recognized in individuals homozygous for Met at codon 129 in the *PRNP *gene [37]. However, Peden et al. reported a case of a patient who was heterozygote at codon 129 of PRNP, suggesting that susceptibility to vCJD infection is not confined only to the Met homozygous PRNP genotype [38]. The human genotype at codon 129 of the *PRNP *gene is known to be a key determinant in human TSEs. This polymorphism modulates phenotype and disease susceptibility to acquired or sporadic prion infection [39]. The large majority of individuals affected by prion diseases are homozygous at codon 129 for either Met or Val [19, 20, 40]. The prevalence of Met/Met is only 39% in the normal Caucasian population, whereas the frequency for Met/Val is about 50% and for Val/Val 11% [19]. In some reports the protective effect of *PRNP *codon 129 heterozygosity is seen in some of the inherited prion diseases [41, 42].

### 2.5. GSS

**Figure 1:**
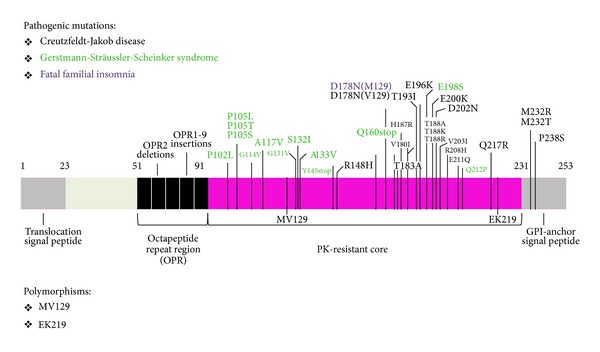
Pathogenic mutations and polymorphisms in the human PrP. The pathogenic mutations associated with human prion diseases are shown above the human PrP coding sequence. These consist of 1, 2, or 4–9 octapeptide repeat insertions (OPR1-9) within the octapeptide repeat region between codons 51 and 91, a 2 octapeptide repeat deletion (OPR2), and various point mutations causing missense or stop amino-acid substitutions. Point mutations are designated by the wild-type amino acid preceding the codon number, followed by the mutant residue, using single letter amino-acid nomenclature. Polymorphic variants are shown below the PrP coding sequence.

GSS is a rare form of prion disease and occurs at a rate of one per 100 million people per year worldwide [43]. In contrast to CJD, GSS is almost always described in a familial context. Only a few sporadic cases resembling GSS have been reported so far [44]. The syndrome was first described in 1928 by the Austrian neurologist Josef Gerstmann (1887–1969), followed by a more detailed report in collaboration with his colleagues Ernst Sträussler and Ilya Scheinker [45]. Most patients show the first symptoms in the fourth or fifth decade of life. Investigations have shown that missense mutations are present in the *PRNP *gene of GSS patients. To date, a variety of mutations have been identified, and the most common is at codon 102 (P102L) [46]; others reported are 105 (P105L) [47], 114 (G114V), 117 (A117V) [48], 131 (G131V) [49], 180 (V180I), 187 (H187R), 198 (F198S) [50], 202 (D202N), 212 (Q212P), and 217 (Q217R) [42]. The STOP mutations reported are Y145STOP-129 M [51, 52], Q160STOP, Y226STOP, and Y227STOP [53] (Figure 1). In addition, several insertional mutations have been described that occur in the N-terminal octapeptide repeat region of PRNP [54, 55].

### 2.6. FFI

Fatal familial insomnia (FFI) was first described in 1986 in a 53-year-old man [56]. Since then, it has been reported in several European countries [57–59], Australia [60], and Japan [61]. The occurrence of FFI is associated with the same codon 178 mutation (D178N) also observed in a subtype of familial CJD [62]. The phenotype caused by the D178N mutation depends on a polymorphism at codon 129. The Met 129-Asn 178 allele segregates with FFI, while the Val 129-Asn 178 allele segregates with fCJD [25]. Recently, the first cases of a sporadic form of fatal insomnia (sFI) have been reported in a 44-year-old man and a 58-year-old woman [63–65]. FFI and sFI have similar disease phenotypes. Both disorders have clinical features of disrupted sleep (loss of sleep spindles, slow-wave sleep, and enacted dreams during rapid-eye-movement sleep), autonomic hyperactivation, and motor abnormalities (myoclonus, ataxia, dysarthria, dysphagia, and pyramidal signs). PET shows pronounced thalamic and limbic hypometabolism that become more widespread in later stages. Neuropathological assessment reveals severe neuronal loss and astrogliosis of the anterior medial thalamus and inferior olives, with later cerebral cortical and cerebellar involvement [66].

### 2.7. Kuru

Kuru (“trembling with fear”) is the prototype of human spongiform encephalopathy. It is restricted to the Fore people living in the Eastern Highlands of New Guinea, where prions were transmitted by ritualistic cannibalism [67]. The disease occurred mostly in children and women, because they consumed the brain of deceased family members. In 1959, the local government banned the cannibalistic practice.

## 3. Prion Conversion: The “Protein-Only” Hypothesis

The central molecular event in the replication of mammalian prions is the self-propagating conformational conversion of PrP^C^ to the misfolded PrP^Sc^ form. This postulate is known as the “protein-only hypothesis” [14]. In recent decades several efforts have been made to understand the mechanism of PrP^C^ to PrP^Sc^ conversion. Two models have been proposed, known as (i) template-directed refolding model and (ii) seeded-nucleation model.

**Figure 2:**
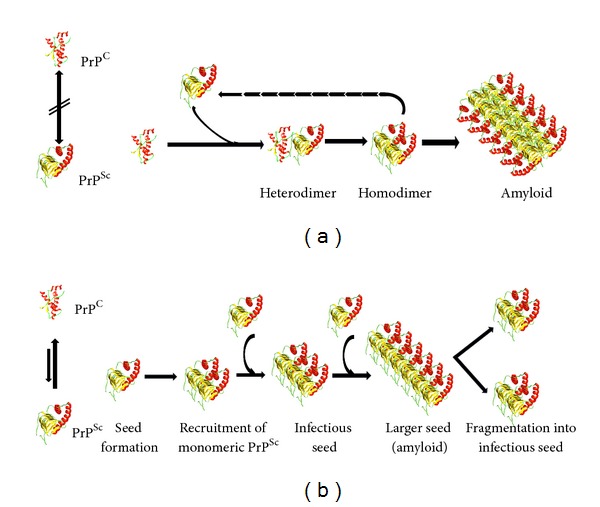
(a) The “template-assistance model” [8] and (b) the “seeding nucleation model” [9].

- The template-directed refolding model postulates a direct interaction between PrP^Sc^ and PrP^C^, which is induced to convert into more PrP^Sc^. A high-energy barrier might prevent the spontaneous conversion of PrP^C^ to PrP^Sc^. In this model the critical step in the conversion is the formation of a dimer between PrP^Sc^ and PrP^C^ or a partially destabilized folding intermediate of PrP^C^ denoted by PrP*. Eventually PrP^Sc^ acts as a template that catalyzes the refolding of PrP^C^ to a thermodynamically more stable PrP^Sc^ conformation (Figure 2(a)).
- The “seeding” or nucleation-polymerization model states that PrP^C^ and PrP^Sc^ are in a reversible thermodynamic equilibrium. So, only if several monomeric PrP^Sc^ molecules (less stable than PrP^C^) are mounted in a highly ordered seed can more monomeric PrP^Sc^ be recruited and eventually aggregated to form amyloid. In such a crystal-like seed, PrP^Sc^ becomes stabilized. The rate-limiting step in this mechanism is not the conformational conversion itself but the nucleation step. Fragmentation of PrP^Sc^ aggregates increases the number of nuclei, which can recruit more PrP^Sc^ and thus seems to replicate the agent. In sporadic prion diseases, fluctuations in the local PrP^C^ concentration might (exceptionally rarely) trigger spontaneous seeding and self-propagating prion replication (Figure 2(b)).

In this transition, the primary structure of PrP does not change, but the secondary and tertiary structures in PrP^Sc^ are considerably different from those in PrP^C^.

### 3.1. Physiological Functions of PrP^*C*^

It is still unclear whether the toxicity of PrP^Sc^ represents a gain of function [68] or whether loss of function of PrP^C^ is responsible for neuropathological changes induced by prions [69]. One thing is certain—PrP^C^ has to be expressed in CNS to permit the conversion into PrP^Sc^, since the infection of PrP-deficient mice, *Prnp* ^0/0^ [Zürich I] [70] or *Prnp* ^−/−^ [Edinburgh] [71], was not successful. As predicted by the protein-only hypothesis, these mice were entirely resistant to prion infections [72]. The ubiquitous presence of PrP^C^ supports the notion that PrP has a generalized cellular function in brain tissue. Several experimental studies [73–75] suggest that PrP^C^ could play a role in synaptic structure, function, and maintenance. Defining the function of PrP^C^ remains one of the main challenges in prion biology, and it is an absolute requirement also for comprehending TSEs attributed to the posttranslational PrP^C^ to PrP^Sc^ conversion.

The focus of this review is not the physiological form of PrP, rather the pathologic PrP^Sc^ scrapie isoform.

## 4. Diagnosis of TSEs

**Figure 3:**
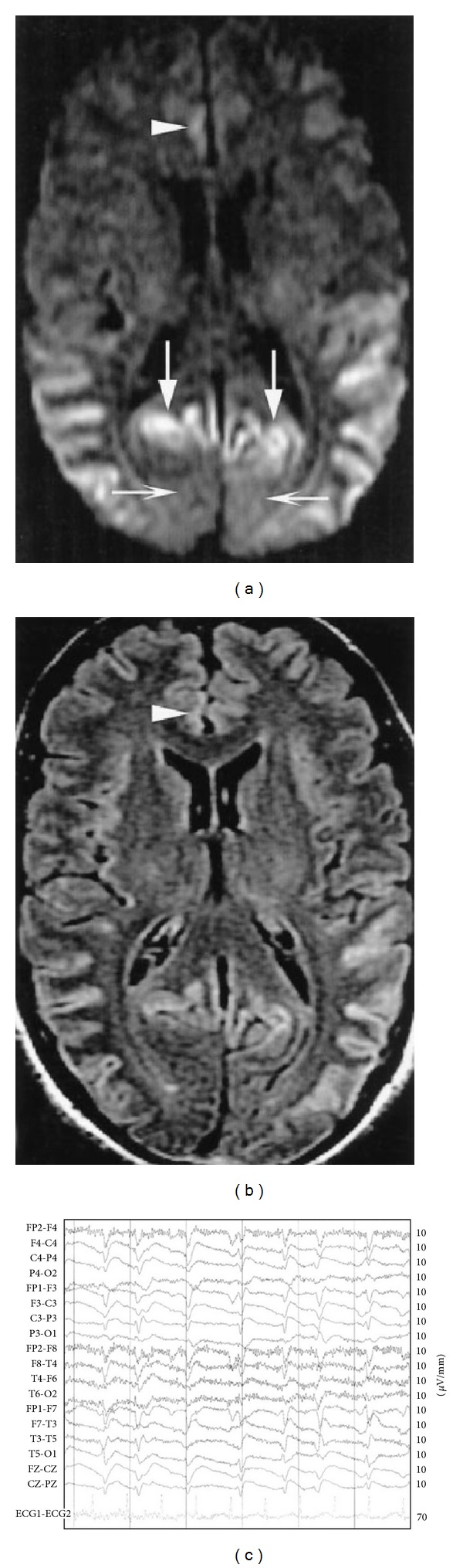
(a) A 50-year-old man with definite sCJD. Axial DWI shows pathologic hyperintensity in bilateral posterior temporoparietal neocortex. Cortex along parietal-occipital fissure is abnormally hyperintense (vertical arrows), but primary visual region is spared (horizontal arrows). Note asymmetric abnormal hyperintensity in right cingulum (arrowhead). Striatum is uninvolved. (b) FLAIR image at same level shows more subtle pathologic hyperintensity in all abnormal regions on DWI, as shown in cingulate cortex (arrowhead) [10]. (c) Definite sCJD (MM1); total duration: 10 months; EEG at 6 weeks: typical (used to classify case as probable); source: http://www.eurocjd.ed.ac.uk.

Unfortunately, confirming a clinical diagnosis of TSEs has historically been difficult, as conventional laboratory tests have been ineffective in detecting them. For example, the cerebrospinal fluid most often appears normal, except for an increase in tau and 14-3-3 proteins. Both of these biomarkers support the CJD diagnosis with a sensitivity of 92% and specificity of 71% [76]. Brain MRI is increasingly useful in identifying sCJD cases. High signal abnormalities in the basal ganglia and/or cortical ribbon on diffusion weighted imaging (DWI) and fluid attenuated inversion recovery (FLAIR) sequences have recently been added to the diagnostic criteria for probable sCJD (Figures 3(a) and 3(b)) [77]. Moreover, neuroimaging with MRI is useful to exclude other causes of subacute neurologic illnesses. Generally, few imaging abnormalities are seen, for example, generalized atrophy in some cases; in less than 10% of sCJD cases, hyperintensity of the basal ganglia may be seen in T2-weighted images. In vCJD, putaminal hyperintensity on T2-weighted images is a common finding [78]. In FFI, PET may detect thalamic hypometabolism although in other prion diseases PET generally shows nonspecific cortical hypometabolism. A helpful test is the electroencephalogram (EEG), which measures brain wave activity (Figure 3(c)). The EEG often shows a characteristic abnormal pattern, typically observed in later stages of the disease, but this technique does not confirm a TSE diagnosis. A definite diagnosis of prion disease, as with any dementia, can be made only by pathologic confirmation following biopsy or autopsy. Since the definitive antemortem detection of PrP^Sc^ in biopsy specimens is discouraged, because it is invasive and poses risks to health care personnel, unfortunately the last option is autopsy and the analysis of postmortem tissue of infected patients [79].

Prion diseases are generally characterized by widespread neurodegeneration and therefore exhibit clinical signs and symptoms of cognitive and motor dysfunction. In addition, infectious prions propagate by forming amyloid plaques, which are considered as the main hallmark of the disease and serve as a main diagnostic criterion. Since PrP^Sc^ is partially resistant to digestion with proteinase K (PK), this characteristic feature has been used to identify infected samples. Other biochemical characteristics useful to differentiate PrP^C^ from PrP^Sc^ are insolubility in nonionic detergents and high content of *β*-sheet secondary structure. The assays for the detection of PrP^Sc^ test brain tissue, where the greatest concentrations of prions are found during the terminal stage of disease. Standard histopathological and immunohistochemical techniques are used to view the tissue microscopically and identify characteristic vacuoles, plaques, or other abnormal features and staining associated with prion diseases. The standard confirmatory test is the Western blot after PK digestion.

### 4.1. Western Blot

**Figure 4:**
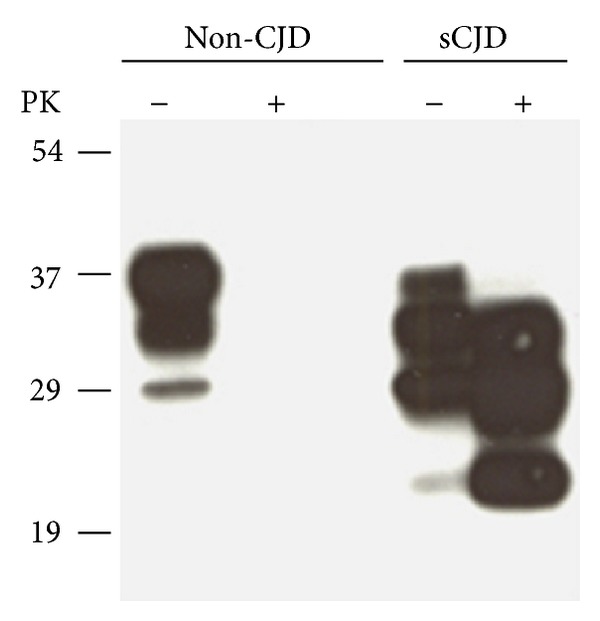
Detection of PK-sensitive PrP^Sc^. (a) Conventional Western blot of PrP treated with or without PK. No PrP was observed after PK treatment in the samples from non-CJD. The PK-resistant PrP27–30 was indicated in the sample from sCJD. Samples were digested with 50 *μ*g/mL proteinase K for 1 hour at 37°C, completely hydrolyzing PrP^C^. Proteinase digestion cleaves ~90 amino acids from the amino terminus of PrP^Sc^ to generate PrP27–30. Blot is developed with anti-PrP mouse monoclonal antibody 3F4 [11].

This technique takes advantage of the partial PK resistance of the scrapie form. Treating PrP^Sc^ with PK results in the removal of only 90 amino acids from the N-terminus (Figure 1). The remaining PrP “core” is denoted by PrP^RES^ (PrP, proteinase resistant). Limited protease digestion of PrP^Sc^ often produces a smaller, protease-resistant molecule of approximately 142 amino acids, referred to as PrP 27–30. Under the same conditions, PrP^C^ and some forms of PrP^Sc^ are completely hydrolyzed. Although resistance to limited proteolysis has proven to be a convenient tool for detecting PrP^Sc^, not all PrP^Sc^ molecules are resistant to protease digestion (denoted by sensitive PrP^Sc^, sPrP^Sc^) [80–84].  Figure 4 shows the typical Western blot profile of infected/uninfected and PK digested/PK not digested brain homogenates.

### 4.2. Conformation-Dependent Immunoassay (CDI)

Another test useful for the detection of prions is the conformation-dependent immunoassay (CDI). This diagnostic test simultaneously measures specific antibody binding to denatured and native forms of PrP [82]. In 1998, Prusiner et al. described this assay as not only able to measure very low levels of PrP^Sc^ but also capable of discriminating among a wide variety of prion strains. In 2002, the same authors [85] reported that CDI is capable of measuring the disease-causing isoform (PrP^Sc^) in bovine brainstems with sensitivity similar to that of the end-point titrations in transgenic (Tg) mice expressing bovine PrP (BoPrP). Prion titers were ~10^7^ ID_50_ units per gram of bovine brainstem when measured in Tg BoPrP mice, a figure ~10 times greater than that determined by bioassay in cattle and ~10,000 times greater than that determined by bioassay in wild-type mice. This immunoassay provides important information about the tertiary and secondary structure of PrP^Sc^, which is strain dependent. Results from CDI should be correlated with those from optical spectroscopic techniques such as time-resolved fluorescence spectroscopy (FRT) and circular dichroism (CD) spectroscopy. The ability to assay features of the tertiary and secondary structure of PrP^Sc^ in crude homogenates opens several new areas of investigation, including determination of PrP^Sc^ structure in various tissues as well as in different regions of the CNS for a variety of prion strains. In 2005, the sensitivity of the assay was improved by selectively precipitating the PrP^Sc^ with Na_2_H[PW_12_O_40_] [86].

### 4.3. Protein Misfolding Cyclic Amplification (PMCA)

As reported by several groups, sustained propagation of PrP^Sc^ (largely in the CNS) results in the accumulation and deposition of the pathogenic protein. Therefore, the conversion into PrP^Sc^ can be reproduced *in vitro* using a technique named protein misfolding cyclic amplification (PMCA) which was pioneered by Soto and colleagues [87]. PMCA allows propagation of PrP^Sc^ *in vitro* from very small amounts of undetectable seeding material to quantities sufficient for detection by Western blot or plate-based immunoassays. For example, using brain-derived PrP^C^ as a substrate, as little as 1 *μ*g/mL of PrP^Sc^ can be detected [88]. This ultrasensitive method has been previously applied to identify prions in a wide range of tissue and fluids from scrapie-infected sheep (blood, feces, saliva, and milk) where only small amounts of the infectious agent reside [89–93]. Given its unique ability to detect prions in readily accessible tissue and at preclinical stages of the disease, PMCA is a viable preclinical test for prion diseases.

### 4.4. Amyloid Seeding Assay (ASA)

Back in 2004, Legname et al. [94] reported the production of synthetic prions via *in vitro *conversion of recPrP [94]. Under different conditions they were able to obtain two different forms of *β*-sheet enriched structures (*β*-oligomer PrP^Sc^-like and recPrP aggregates in fibrillar amyloid form). The polymerization process was monitored by simply applying thioflavin (ThT) to the reaction mixture. This dye shows strong increase of fluorescence upon binding to *β*-sheet-rich structures like amyloid aggregates. Importantly, in this work authors discovered that the addition of a seed of prefolded amyloid to the fresh reaction substantially shortens the fibrillation process (called lag phase). This experiment shows that recPrP fibrils can be induced by seeding, defining the technique as amyloid seeding assay (ASA). Later in 2007, the authors reported that the ASA detected PrP^Sc^, the sole component of the prion, in brain samples from humans with sporadic Creutzfeldt-Jakob disease as well as in rodents with experimental prion disease [95]. Using the ThT assay, they found that many prion strains are capable of seeding the polymerization of recPrP into amyloid, demonstrating that this seeding property can be used as an assay to detect prions in biological samples [95].

### 4.5. Real-Time Quaking-Induced Conversion Assay (RT-QUIC)

The development of *in vitro* techniques, such as PMCA and ASA, has generated the potential for sensitive detection of prions. Quaking-induced conversion assay (QUIC) is another PrP^Sc^ amplification assay similar to ASA [96]. This *in vitro *PrP^Sc^ amplification technique employs soluble recombinant PrP (rPrP-sen) as a substrate, which is seeded with PrP^Sc^ and then subjected to intermittent automated shaking. This technique can be performed more easily than PMCA, which requires repeated sonication. Previous studies have shown that QUIC assays correctly discriminate between normal and scrapie-infected CSF samples in both hamster and sheep prion disease models [97, 98]. More recently, a more refined QUIC assay, known as real-time quaking-induced conversion assay (RT-QUIC), was designed [99]. RT-QUIC offers sensitivity similar to the *in vivo *bioassay in hamsters but is roughly 50–200 times faster and much less expensive. RT-QUIC allows the detection of ≥1 fg of PrP^Sc^ in diluted Creutzfeldt-Jakob disease (CJD) brain homogenate [99]. These findings indicate the promising enhanced diagnostic capacity of RT-QUIC in the antemortem evaluation of suspected CJD [100]. Moreover, Gmitterová et al. reported that the ELISA assay, which measures all 14-3-3 isoforms, was very useful in PrP^Sc^ detection [101]; however, this system is not commercially available. Therefore, according to the World Health Organization, diagnosis of prion diseases is usually based on medical history, symptoms (myoclonus, depression), and diagnostic tests, for example, MRI scans and EEGs.

## 5. Compounds That Target **PrP^Sc^**

The presence of PrP^Sc^ deposits is considered a hallmark for prion diseases and serves as a main diagnostic criterion. At the same time it represents a therapeutic target for pharmacological intervention. In fact, treatment investigations target mostly the accumulation of PrP^Sc^ in the brain. Dozens of drug candidates for TSEs have been reported to date, but only very few proved to be effective in* in vivo* studies. The two most promising compounds, quinacrine and pentosan polysulphate, have largely been dismissed as ineffective in patients [102, 103]. A number of compounds have shown antiprion activity in numerous studies using prion inhibitory assays in cell culture [104–107]. These compounds include sulfated polysaccharides, for example, pentosan polysulphate [108], Congo red and other azo dyes [109], amphotericin B and analogues [110], anthracyclines [111], phthalocyanines and porphyrins [112], phenanthridine derivatives [113], inorganic ions, branched polyamines, antagonists of the N-methyl-D-aspartate receptor, such as memantine [114], and acridine derivatives, such as quinacrine [115–117]. Immunotherapeutic approaches are also being attempted for prion infection, with various levels of success [106, 118, 119]. In addition, further methods have recently been reported in the screening of large compound collections *in vitro* [113, 120, 121]. 

## 6. Diagnosis of Alzheimer's Disease

Whereas prion diseases are a rare form of neurodegenerative diseases leading to dementia, Alzheimer's disease (AD) is the most common one. 

The pathological features of AD include neuritic plaques composed of amyloid-*β* peptide (A*β*) fibrils, neurofibrillary tangles of hyperphosphorylated tau (NFT) protein, and neurotransmitter deficits. Although there has been a rapid increase in the understanding of the etiology, genetics, and underlying pathophysiological mechanism for AD during recent years, there is still no cure for the disease. Therapy is mainly symptomatic as it aims to replace the neurotransmitter deficits. In the quest for disease-modifying treatments, many drug development programs pursue strategies directly related to amyloid or tau. Indeed, these extracellular plaques and deposits of A*β* and intracellular NFT became over the years the pathological hallmark of AD and drug targets. Despite a robust support for the importance of both, most efforts have focused so far on developing antiamyloid agents to be used in the early stages of the disease. A prerequisite for the early treatment of the disease would be early detection of AD plaques. Therefore, several strategies have been developed for the imaging of amyloid, namely, radiolabeled amyloid-*β* peptide (A*β*) antibodies and peptide fragments, small molecules for PET and SPECT imaging, and compounds for MRI. 

Several research groups have adopted the small-molecule approach to develop substances suitable for amyloid imaging. Some of the most promising compounds are derivatives of Congo red, thioflavin T, stilbene, and FDDNP. Some of them, like [^18^F]FDDNP and [^18^F]TZDM, have been reported to have affinity for diffuse plaques or A*β*_1–42_-positive plaques [122, 123]. Notably, FDDNP has been reported to label also PrP plaques in brain sections [124]. However, these compounds have some limitations in their practical use as probes for *in vivo* imaging, because of their delayed washout and nonspecific accumulation in the brain white matter [125]. Nonspecific binding of imaging probes leads to high background activity and low contrast images of target structures, resulting in difficult early detection of plaque deposits. Therefore, some basic criteria need to be followed to obtain a small-molecule probe for amyloid plaques (Table 1). Table 1 lists the criteria of an ideal imaging compound for the detection of amyloid in brains of living patients with AD.

The visualization of amyloid plaques in the brains of living patients with AD would greatly aid the assessment of efficacy for antiamyloid therapy. To date, a number of groups have worked on MRI [126, 127] and PET [12, 128, 129] probes for amyloid plaques. Notably, the PET ligand Pittsburgh compound B ([^11^C-]PIB, or 6-OH-BTA-1) has shown promise in early clinical trials and is currently used in a number of human studies [130, 131]. Other groups reported the development of the new near infra-red fluorescent (NIRF) ligands for A*β* [132, 133]. Due to the short physical half-life of carbon-11 (20.4 minutes), recently, great efforts have focused on the development of A*β* plaques tracers radiolabeled with fluorine-18, a radioisotope with a considerably longer half-life (109.4 minutes). Some of them, like 4-(N-methylamino)-4′-(2-(2-(2-[^18^F]fluoroethoxy)ethoxy)ethoxy)-stilbene ([^18^F]BAY94-9172, florbetaben, with Ki = 2.22 ± 0.54 nM) [1, 2] and 2-(3-[^18^F]fluoro-4-methylaminophenyl)benzothiazol-6-ol ([^18^F]GE-067, flutemetamol, Ki = 0.74 ± 0.38 nM) [3], had already been reported under clinical trials. In April 2012, (E)-4-(2-(6-(2-(2-(2-[^18^F]fluoroethoxy)ethoxy)ethoxy)pyridin-3-yl)vinyl)-N-methylaniline ([^18^F]AV-45, florbetapir, Ki = 2.87 ± 0.17 nM) [134, 135] had been approved by the US Food and Drug Administration (FDA) as a radioactive diagnostic agent indicated for brain imaging of A*β* plaques in patients who are being evaluated for AD and other causes of cognitive impairment. Although autopsy remains the only positive way to diagnose Alzheimer's disease, being able to identify the A*β* plaques *in vivo* is a major step forward.

Because the biologic role of *β*-amyloid peptides is uncertain, researchers are also investigating alternative targets of intervention at various stages of progression. Ongoing efforts by the research community to qualify biomarkers in clinical trial designs and methods for enriching study populations with patients with early-stage Alzheimer's disease reflect important FDA priorities. Despite our growing understanding of the relationship between various disease-based biomarkers and the clinical course of Alzheimer's disease, it remains unclear whether the effect of a drug on one or more such biomarkers can actually predict a meaningful clinical benefit.

## 7. Amyloid Dyes and Their Derivatives

As mentioned above, candidate probes have primarily been derived from amyloid dyes such as Congo red (CR) and thioflavin T (ThT) [125]. Among all amyloid-staining compounds, CR provides historically the most standardized way of staining amyloid plaques and is still employed in postmortem histological analysis of AD brains, as the binding is specific [136]. Here we review a few aspects of Congo red, thioflavin T, and their derivatives.

### 7.1. Congo Red (CR)

**Scheme 1:**
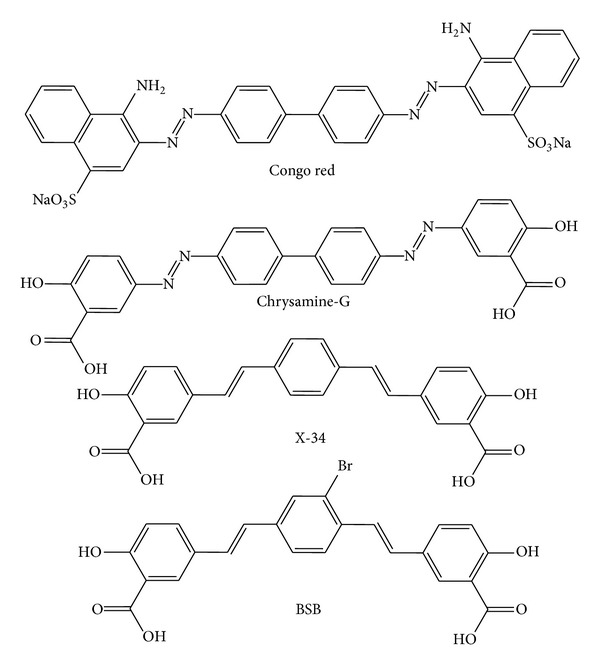
Chemical structures of Congo red and its derivatives, potential *in vivo* imaging agents for *β*-amyloid plaques.

Congo red (Scheme 1) was invented in 1884, by the young German chemist Paul Böttiger (Böttiger, P. Deutsches Reichs Patent 28753, August 20, 1884). He created the first “direct” dye that did not require additional substances for fixation to the textile fibers [137]. The mechanism of interaction of CR with amyloid fibrils is not well understood. Some studies suggest that the origin of the specific binding of CR to amyloid-*β* aggregates is due to the combination of electrostatic interactions between the negatively charged CR's sulfonate groups with the positively charged amino-acid residues in the *β*-sheet structures [138, 139]. However, it is even generally believed that CR's binding depends on the secondary configuration of the fibril, consisting predominantly of cross-*β*-sheets [140]. Unexpectedly, recent investigations indicate that the dye also possesses the capacity to interfere with processes of protein misfolding and aggregation. This is possible by stabilizing native protein monomers or partially folded intermediates, while reducing the concentration of more toxic protein oligomers [141]. In fact, CR is able to block A*β* aggregation and toxicity in rat hippocampal neuron culture [142, 143], in HeLa and PC12 cells [144], and in human macrophage culture [145]. Although the effect of CR in transgenic mouse models of AD has not been investigated so far, CR exerted a positive effect on other experimental models, such as *Drosophila melanogaster*. Feeding with 5% w/v CR from the embryonic stage resulted in marked survival prolongation, and further histological analysis showed the reduction in the amount of A*β* aggregates and preservation of brain and retinal tissue [146]. Back in 1992, Caughey and Race [147] reported that CR suppresses even PrP^Sc^ accumulation and inhibits scrapie agent replication (in interval going from 1.4 *μ*M to 42 *μ*M) in cell culture studies (on mouse neuroblastoma cells, N2a), showing that the accumulation of PrP^Sc^ remained suppressed even after CR removal. In *in vivo *studies, CR has been observed to exert an ameliorative effect in animals experimentally infected with two different prion strains (263K and 139H) [148, 149]. Dosages of 0.1 and 10 mg CR (i.p.) did not have any effect, while higher CR dosages (10 mg once a week or 5 mg twice a week) induced a small increase in incubation time in i.c. scrapie-infected mice. A cumulative weekly dose of 75 mg of CR distributed over six days (12.5 mg) had a considerable effect, with incubation times extended almost to 14 days. In the i.p. infected animals, the lower dosages of 1 mg and 10 mg of CR produced a similar extension of incubation time. In a second trial of the same study, CR in dosages of 25 mg per day was given over 6 days, 1 or 2 weeks before inoculation, at the day of infection, or 1, 2, 3, or 4 weeks later. The maximal effect was achieved if treatment was initiated on the same day of scrapie infection. Treatments started 2 weeks before or 2 weeks after infection were less effective and almost ineffective if started at 3 and 4 weeks after infection. Thus, the timing of CR treatment is crucial for beneficial effect. On the other hand, other *in vitro* experiments, either with A*β* [150] or PrP^Sc^ [151], showed that at low concentrations CR can promote the protein aggregation. Hence, the effect of CR on fibril formation can be either inhibitory or stimulatory depending on its concentration. At low concentrations, CR binding populates generation of partially folded, aggregation-prone forms of proteins (oligomers and protofilament intermediates) resulting in accelerated fibril formation. At higher concentrations, however, CR inhibits fibril arrangement supporting the denatured state, which is much less prone to aggregation. Since CR is toxic (highly carcinogenic due to its benzidine structure) and is not able to cross the BBB, derivatives have been developed and made suitable for antemortem and *in vivo* visualization and quantification of brain amyloids. Here we report some examples of CR derivatives able to inhibit some of these aggregated proteins. Chrysamine-G, X-34, and BSB were the most promising derivatives of CR dye.

### 7.2. Chrysamine-G

Chrysamine-G (CG) is the most intensively examined compound among structural analogues of CR. In this derivative, naphthalenesulfonic acid groups are exchanged for salicylic acid groups with a retained interdistance of 19–20 Å. Its smaller size, as compared to CR, and the higher lipophilicity allow it to cross the intact BBB when injected in a dose of 1 mg/kg in mice [139]. Most importantly, CG appears to be less toxic than CR, since the administration *in vivo* (10 mg/kg–100 mg/kg via i.p.) did not induce any notable behavioral effects in mice during an observation period of up to 72 h [139]. When incubated with human postmortem brain tissue homogenates, [^11^C]CG showed the labeling of amyloid angiography and significantly higher binding in the frontal, temporal, and parietal cortices of AD patients in comparison to those of age-matched controls [152]. CG appeared to be a more potent A*β* inhibitor than CR, with effective concentrations of the latter being in the range of 2–20 *μ*M. This finding is in agreement with higher binding affinities of CG than CR to synthetic A*β* (Ki of 0.37 *μ*M and 2.8 *μ*M, resp.). Chrysamine-G even attenuated A**β**_25–35_)-induced toxicity in PC12 cells, validated as a decrease in MTT reduction in the concentration range of 0.2–2 *μ*M [153].

### 7.3. X-34

X-34 (1,4-bis-(3-carboxy-4-hydroxyphenylethenyl)-benzene) is a highly fluorescent CG derivative, whose structure consists of a central benzene ring, where the two diazo bonds (N=N) were replaced by alkene bonds (C=C). Most importantly, naphthalenesulfonic acids of CR are substituted by salicylic acids; as for CG, this change results in higher lipophilicity and better BBB penetration capacity. This compound has shown promising staining properties of the *β*-sheet structures of amyloid plaques and cerebrovascular amyloid in AD autopsy of brain tissue [154].

### 7.4. BSB

In 2000, Skovronsky and colleagues reported the synthesis of another CR derivative, BSB [(trans, trans)-1-bromo-2,5-bis-(3-hydroxycarbonyl-4-hydroxy)styrylbenzene], demonstrating its high binding affinity for A*β* aggregates *in vitro* (Ki = 0.4 *μ*M) [155]. Like X-34 and CG, BSB specifically labels senile plaques in postmortem AD brain sections. The authors even observed that BSB permeates living cells in culture and binds specifically to intracellular A*β* aggregates. After i.c. injection in living transgenic mouse models of AD amyloidosis, BSB labels plaques composed of A*β* with high sensitivity and specificity. Lastly, BSB crosses the BBB and labels numerous AD-like plaques throughout the brain of the transgenic mice after i.v. injection. Thus, the authors concluded that BSB is an appropriate starting point for future efforts to generate an antemortem diagnostic tool for AD. In 2004, Ishikawa et al. [156] hypothesized the application of BSB in the prion field. The authors found that BSB bound to compact plaques of PrP^Sc^, not only in the brain specimens of certain types of human TSEs but also in the brains of TSE-infected mice, when the probe was injected intravenously. The compound was also able to inhibit abnormal PrP^Sc^ formation in a cellular model of TSE with IC_50_ value of 1.4 *μ*M. Furthermore, in an additional experimental mouse model, the intravenous injection of 1 mg BSB prolonged the incubation period by 14% [156]. The efficacy was only observed against the RML strain. Hence, this compound is promising not only as imaging probe but also for therapeutic purposes in TSEs caused by certain strains. 

## 8. Thioflavin T (ThT)

**Scheme 2:**
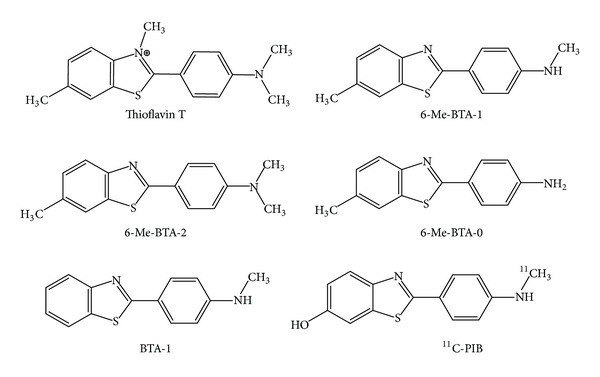
Chemical structures of thioflavin T and its derivatives, useful for potential *in vivo* imaging for *β*-amyloid plaques.

Thioflavin T (ThT) is another dye useful in the analysis of aggregated amyloid proteins (Scheme 2), and it is widely used even to examine fibrillation kinetics *in situ*. In 1959, Vassar and Culling first described the use of the benzathiole dye thioflavin T as a potent fluorescent marker of amyloid in histology [157], demonstrating the potential of fluorescent microscopy for amyloid fibril diagnosis. They noted that ThT is selectively localized in amyloid deposits, thereupon exhibiting a dramatic increase in fluorescent brightness. In fact, the binding to amyloid deposits is slightly weaker than with CR (Ki in the sub- and low *μ*M range), but it exhibits a green fluorescence that becomes more than 1000 times brighter upon binding to amyloid plaques [158]. Afterwards, Naiki et al. and LeVine [158–163] were among the first to characterize the fluorescence spectra and binding properties of ThT. They showed that, upon binding of fibrils, ThT displays a dramatic shift of the excitation maximum (from 385 nm to 450 nm) and the emission maximum (from 445 nm to 482 nm) and that ThT fluorescence originates only from the dye bound to amyloid fibrils [159–161]. These studies showed that dye binding is linked to the presence of the cross-*β* structure of fibrils. However, the lack of an atomic resolution structure of amyloid fibrils complicates the elucidation of the binding mode. Unfortunately, ThT possesses the disadvantage of containing a charged group, the positively charged quaternary nitrogen of the benzothiazolium group (Scheme 2), which would likely limit the permeation of the BBB of this compound. However, the ability of ThT to specifically recognize and bind with modest affinity to amyloid has allowed it to serve as an excellent starting scaffold for derivatization and elaboration to generate a number of amyloid stains and clinical reagents, included for use in medical imaging of amyloid in living patients [12, 164, 165].

### 8.1. 6-Me-BTA-0, 6-Me-BTA-1, and 6-Me-BTA-2

In 2001, Klunk et al. [165] showed that removing the charge from ThT affected the amyloid-binding properties of ThT derivatives. In that work the authors reported the synthesis of three ThT derivatives, 6-Me-BTA-0, 6-Me-BTA-1, and 6-Me-BTA-2, all of which were 600-fold more lipophilic than ThT. They found that the binding to A*β*_1–40_ fibrils presented higher affinity (Ki = 20.2 nM) than ThT (Ki = 890 nM). These uncharged ThT derivatives stained both plaques and neurofibrillary tangles (NFT) in postmortem AD brain, showing some preference for plaque staining. Furthermore, they examined whether an uncharged, lipophilic derivative of ThT would enter the brain in amounts sufficient for imaging by PET. That compound, designed as [N-methyl-^11^C]6-Me-BTA-1, entered the brain at levels comparable to those commonly used by neuroreceptor imaging agents (0.223 %ID-kg/g or 7.61 %ID/g at 2 min after-injection) and showed good clearance of free and nonspecifically bound radioactivity in normal rodent brain tissue (brain clearance *t*_1/2_ = 20 min). In contrast, the 6-Me-BTA compounds did not display the classic shift in excitation and emission spectra when bound to A*β* that has been well documented for ThT.

### 8.2. BTA-1 and 6-OH-BTA-1

One year later, the same group [166] showed that the derivative without the methyl group in position 6 of benzothiazole moiety, denoted by BTA-1 or (2-[4′-(methylamino)phenyl]benzothiazole (Scheme 2), had more promising characteristics than the previously reported compounds. This molecule presented high affinity for the amyloid plaques (Ki = 11 nM for A*β*_1–40_), and the intravenous injection of [^11^C-] labeled BTA-1 in wild type mice resulted in high brain uptake (12.9 %ID/g at 2 min after-injection). Importantly, [^11^C]BTA-1 is characterized by relatively rapid egress of radioactivity from normal brain tissue. Amyloid deposits were imaged with multiphoton microscopy in the brains of living PS1/APP transgenic mice following the systemic injection of unlabeled BTA-1. The authors concluded that the [^11^C]BTA-1 was a promising radioligand for further development as a PET amyloid-imaging agent for AD.

**Figure 5:**
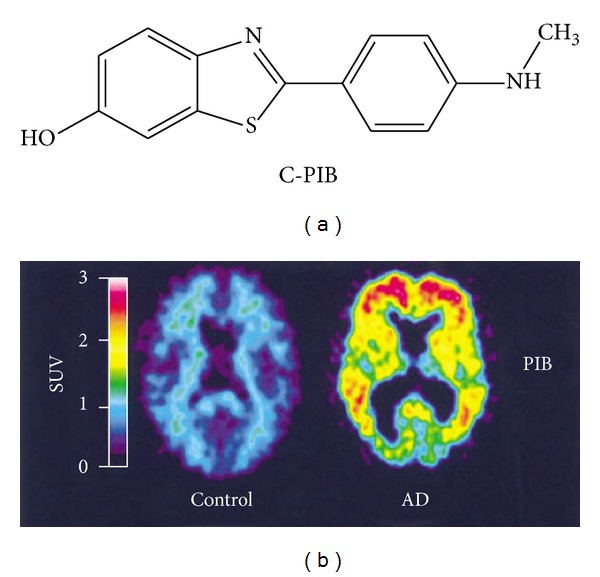
PET imaging of human AD brain using [^11^C-] PIB [12]. PIB standardized uptake value (SUV) images show a marked difference between PIB retention in AD patients and healthy control subjects. PET images of a 67-year-old healthy control subject (left) and a 79-year-old AD patient. The left column shows lack of PIB retention in the entire gray matter of the healthy subject.

In 2004, this uncharged ThT derivative was taken into consideration also by Ishikawa et al. as PrP^Sc^ inhibitor and as a molecule able to label PrP deposition in TSE brains [156]. Using a well-known PrP^Sc^ inhibition assay in cell culture on ScN2a, the authors found that BTA-1 had a promising inhibitory activity (IC_50_ = 4 nM) and low toxicity, since no apparent changes were observed up to 10 *μ*M of treatment. Next, they assessed its utility as diagnostic imaging tool for PrP plaques using the histopathological specimens from human TSE cases. They found that it was able to fluorescently label most of the PrP plaques in the cerebral cortices of GSS cases and of variant CJD cases, whereas it was not able to stain PrP plaques of sporadic CJD cases. Similar results were observed when the postmortem brains of Tg7 mice infected with the 263K strain were used, considering that it stained the plaque type of PrP in the cerebral white matter between cortex and hippocampus. Due to the absence of positive charge and its capability to cross the BBB, they even performed *in vivo* experiments using Tg7 mice infected with 263K strain. A bolus injection of BTA-1 labeled PrP plaques in the white matter between cortex and hippocampus of the affected brains. Faint cerebrovascular labeling was occasionally observed at 4 h after the injection, but not at 18 h or later. Moreover, no significant labeling was observed in uninfected transgenic mice. Similar results were observed in Tga20 mice infected with RML strain, although labeled PrP plaques were less frequently observed. Even the 6-hydroxy BTA-1 derivative (also called PIB or 6-OH-BTA-1) inhibited PrP^Sc^ formation in ScN2a cells with an IC_50_ in the nanomolar range; more importantly, it has been selected for the first human trial of a benzothiazole amyloid-imaging agent [131]. In their latter report, Rowe et al. analyzed 16 patients with diagnosed mild AD and 9 controls. Their results demonstrated that PET imaging with the [^11^C-]PIB tracer provided quantitative information on amyloid deposits in living individuals with AD. Thanks to the favorable radiotracer profile, PIB has become the most commonly used PET amyloid agent, adopted in more than 40 research centers worldwide (Figure 5). However, a recent study highlighted that [^11^C-]PIB PET does not detect PrP-amyloid in prion disease patients, including variant Creutzfeldt-Jakob disease [167].

### 8.3. NIAD-4

**Scheme 3:**
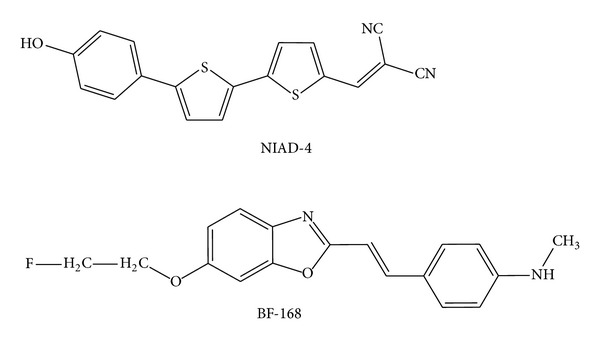
Chemical structures of potential *in vivo* imaging agents for *β*-amyloid plaques.

Another emerging approach for *in vivo* detection of aggregated proteins is optical imaging through special near-infrared (NIR) fluorescent contrast agents. In 2005, following the rules reported in Table 1, Nesterov et al. designed a small molecule known as [[5′-(4-hydroxyphenyl) [2,2′-bithiophen]-5-yl]methylene]-propanedinitrile, or simply NIAD-4 (Scheme 3) [133]. The binding studies with artificially aggregated amyloid protein assays revealed that NIAD-4 binds to the same site as BTA-1 with a Ki of 10 nM. This affinity is much higher than that of ThT (Ki = 580 nM) and is close to that of high-affinity amyloid-binding compounds like PIB (Ki = 4.3 nM) [168]. Nesterov et al. [133] studied the specificity of NIAD-4 binding to A*β* by *in situ* histochemical staining of fixed sections from transgenic mouse brain. Brain sections were obtained from aged APP transgenic mice with AD-like pathology. The brain sections were labeled with a NIAD-4 (10 *μ*M) solution in DMSO/propylene glycol for 15 min at room temperature. *In vitro* fluorescence imaging showed high-specificity labeling of NIAD-4, which revealed the exact position and size of the aggregated A*β* deposits. The authors [133] showed also the *in vivo* A*β* binding of NIAD-4 in aged APP transgenic mice. Mice were prepared with cranial windows to allow direct monitoring of the brain surface and then administered 10 *μ*M of 2 mg/kg NIAD-4 solution by i.v. injection. Red fluorescence imaging using multiphoton microscopy showed that the agent readily crossed the BBB and labeled specifically both the plaques and cerebrovascular amyloid angiopathy. A radiolabeled version of NIAD-4 may also be advantageous for PET or SPECT imaging.

### 8.4. BF-168

Several stilbene derivatives have been synthesized as compounds for the probing of amyloid plaques [169]. Stilbene shows binding to A*β* aggregates in the nanomolar range [170]. Similar series of imaging probes were reported in [171], describing *in vitro* and *in vivo* properties of some styryl-based derivatives of ThT. The most promising one was 6-(2-fluoroethoxy)-2-[2-(4-methylaminophenyl)ethenyl]benzoxazole (BF-168) (Scheme 3). In AD brain sections, BF-168 selectively binds senile plaques and recognizes A*β*_1–42_-positive diffuse plaques as well as neuritic plaques. Intravenous injection of BF-168 in PS1/APP and APP23 transgenic mice resulted in specific *in vivo* labeling to both compact and diffuse amyloid deposits in the brain. In addition, ^18^F-radiolabeled BF-168 intravenously administered to normal mice showed abundant initial brain uptake (3.9 %ID/g at 2 min after injection, a sufficient level for brain imaging probe) and fast clearance (*t*_1/2_ = 24.7 min, indicating fast brain washout) sufficient for the compound to be a PET imaging probe. Furthermore, autoradiograms of brain sections from APP23 transgenic mice at 180 min after intravenous injection of [^18^F]BF-168 showed selective labeling of brain amyloid deposits with little nonspecific binding. These findings strongly suggest that styrylbenzoxazole derivatives are promising candidate probes for PET and SPECT imaging for early detection of amyloid plaque formation in high-risk AD patients in presymptomatic stage [171]. Additionally, this new styrylbenzoxazole compound clearly labeled PrP^Sc^ plaques in brain specimens from human TSEs (sCJD and vCJD) [172]. BF-168 also inhibited abnormal PrP formation in TSE-infected cells with IC_50_ = 0.4 nM in ScN2a cell line model and prolonged the lives (~11.4%) of mice infected intracerebrally with TSE when the compound was administered intravenously at the preclinical stage. Even though their efficacy depends on the pathogen strain, these derivatives are a new class of compounds with potential as both therapeutic drugs and imaging probes for TSEs.

### 8.5. G8

**Figure 6:**
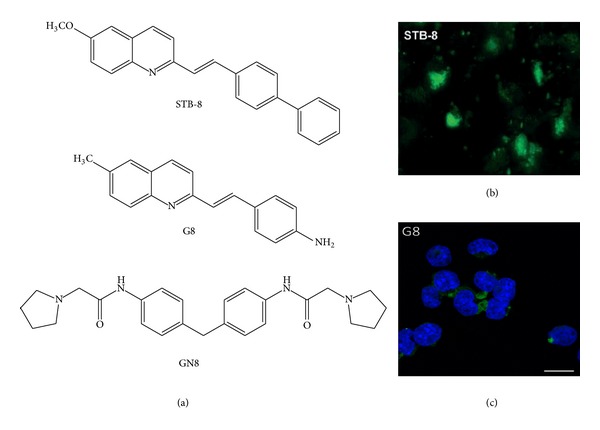
(a) Chemical formula of the three promising antiprionic compounds and *in vitro* staining with (b) STB-8 compound (*β*-amyloid plaques) and (c) G8 compound (PrP^Sc^ deposits).

Meanwhile, other styryl derivatives have been studied [129, 169, 173, 174]. Li et al. [175] tested a group of styryl-based neutral compounds as potential *in vivo* imaging agents for *β*-amyloid plaques. The most promising one in this work was designed as STB-8 (Figure 6(a)), and its use in *ex vivo *and *in vivo *imaging experiments on an AD transgenic mouse model showed excellent BBB permeability and specific staining of the *β*-amyloid plaques (Figure 6(b)) [175]. 

A similar chemical scaffold was reported in our recent work [7]. The compound (E)-6-methyl-4-amino-2-styryl-quinoline or G8 is a small molecule (Figure 6) with the proper features to potentially diagnose, deliver therapy, and monitor response to therapy in protein misfolding diseases. These features include compound fluorescent emission in the NIR region and the ability to interact with both A*β* and prion fibrils, staining them with high selectivity. Moreover, the compound possesses an antiaggregation property against A*β*_1–42_ using the well-known ThT-based fluorimetric assay [176] and prolongs the lag phase of PrP^Sc^ formation in fibrillation assay [177]. At a concentration of 50 *μ*M, G8 delayed fibril formation, extending the lag phase to ≥70 h (control: 59 h). A similar profile was found for GN8, an antiprion drug candidate (Figure 6) for which a specific binding with PrP has been experimentally shown [178]. With such a good* in vitro* profile, we treated the ScGT1 and ScN2a cell lines with the compound, and the viability was quite good. At 1 *μ*M concentration, G8 showed a very low toxicity, with cell viability above 90% if compared with nontreated cells, while at a 10 *μ*M concentration it still showed a tolerable toxicity, with a residual 60% cell viability not different from that of drug candidate GN8. Starting from these nontoxic concentrations, we treated the cells to evaluate their inhibitory activity, and we found that the compound possessed a submicromolar capability to inhibit PrP^Sc^ (EC_50_ = 0.5 ± 0.1 *μ*M), greater than GN8 (EC_50_ = 1.5 ± 0.1 *μ*M) in our system. To confirm the labeling of PrP^Sc^ aggregates in living cells, fluorescent staining with G8 was carried out using the same ScGT1 and ScN2a cell models. We found that 0.025% of G8·HCl (0.84 mM) was sufficient to observe many fluorescent spots in the treated cells examined by fluorescent microscopy (Figure 6(c)). Importantly, no spots were observed in the uninfected cells, confirming a specific binding. Furthermore, the staining pattern was consistent with that observed with 0.025% thioflavin S (ThS), a common PrP^Sc^ dye. A further experiment proved that G8·HCl (0.25 mM) distinguishes the abnormal, aggregated, and PK-resistant PrP^Sc^ isoform from the normal, PK-sensitive PrP^C^ isoform. Thus, after eliminating PrP^C^ through a PK digestion step, the previous fluorescence-staining pattern was observed. We primarily used the FITC filter set for these studies, but we confirmed the staining by employing the ThS one, which is within the NIR optical window. G8 was able to cross the BBB in an *in vitro* model, such as parallel artificial membrane permeability assay (PAMPA, Pe 23.1 ± 1.9 10^−6^ cm^−1^).

From a medicinal chemistry perspective, G8 offers peculiar advantages: (1) a lower molecular weight than previous sensors [179] and (2) a small-molecule scaffold that is easily amenable to further manipulation to improve fluorescence response and amyloid-binding properties. Most importantly, with respect to the previously reported NIR amyloid sensors [132, 133, 180–182] it offers the advantage of a concomitant promising antifibrillar profile (*in vitro* and in a cellular context), together with a low toxicity. If these distinctive properties are confirmed *in vivo*, G8 is likely to become the first purposely designed therapeutic and diagnostic (theranostic) tool for prion diseases and AD.

## 9. Conclusion

All the efforts made to date to develop rapid, accurate, and highly sensitive antemortem tests to detect prions early in the course of the disease have failed. Most tests still involve PK digestion, and the specificity and sensitivity of tests that do not use PK require further validation. Nevertheless, neuroimaging shows promise as a future clinical diagnostic tool for neurodegenerative diseases. Continued expansion of scientific imaging tools has been essential toward a new standard strategy that links established *in vitro* and cell culture experimental assays to imaging studies for living subjects. In fact, over the last few years the rapid development of different compounds suitable for visualizing aggregated *β*-sheet-rich proteins has led to the first promising *in vivo* studies of the amyloid ligands, such as PIB [12]. Florbetapir is the first radioactive dye for brain imaging of amyloid plaques to be approved by the FDA. With its introduction into the clinical practice, we are now effectively entering the era of neurodegenerative disease imaging.

Our hope is that our own G8 molecule [7] will confirm *in vivo* the results obtained *in vitro*. Molecular imaging in living subjects offers distinct advantages when compared with conventional *in vitro* and cell culture research techniques in biology. Therefore further work on promising imaging compounds is necessary to access *in vivo* studies. The use of these compounds could represent a good approach to detect and treat neurodegenerative disorders such as Alzheimer's disease and prion diseases. As the term theranostics is derived from the words therapeutics and diagnostics, the final application of theranostics is combining disease diagnosis and therapy. This combination in a single molecule enables real-time feedback on the biodistribution and the target site accumulation of the compound. The concurrent delivery and readout of efficacy can be exploited to tailor treatment regimens for specific treatment groups. 

Effective treatments for devastating disorders such as Alzheimer's disease and prion diseases are urgently needed, as the world's population continues to age. We are confident that purposely-designed theranostics might soon become powerful tools to combat them.

## Figures and Tables

**Table 1:** Ideal properties for a diagnostic small molecule.

(i) Stable *in vivo *	
(ii) Moderately lipophilic	
(iii) Entering the brain in sufficient amounts and retained in the brain	
(iv) Low uptake of metabolites to brain	
(v) Detection of plaques (imaging properties)	
(vi) High specificity for amyloid deposits, low nonspecific bonding	
